# Exploring brain plasticity in developmental dyslexia through implicit sequence learning

**DOI:** 10.1038/s41539-024-00250-w

**Published:** 2024-05-27

**Authors:** Gaia Olivo, Jonas Persson, Martina Hedenius

**Affiliations:** 1 University of Gothenburg, Department of Psychology, Haraldsgatan 1, 405 03 Göteborg, Sweden; 2 Karolinska Institute, Department of Neurobiology, Care Sciences and Society, Aging Research Center, Tomtebodavägen 18a, SE-171 65 Solna, Sweden; 3 Center for Life-span Developmental Research (LEADER), School of Law, Psychology, and Social Work, Örebro University, Örebro, Sweden; 4 Uppsala University, Department of Public Health and Caring Sciences, Biomedical Center, Husargatan 3, 751 22 Uppsala, Sweden; 5 Center of Neurodevelopmental Disorders (KIND), Centre for Psychiatry Research; Department of Women’s and Children’s Health, Karolinska Institutet & Stockholm Health Care Services, Region Stockholm, Gävlegatan 22, 11330 Stockholm, Sweden

**Keywords:** Learning and memory, Dyslexia

## Abstract

Developmental dyslexia (DD) is defined as difficulties in learning to read even with normal intelligence and adequate educational guidance. Deficits in implicit sequence learning (ISL) abilities have been reported in children with DD. We investigated brain plasticity in a group of 17 children with DD, compared with 18 typically developing (TD) children, after two sessions of training on a serial reaction time (SRT) task with a 24-h interval. Our outcome measures for the task were: a sequence-specific implicit learning measure (ISL), entailing implicit recognition and learning of sequential associations; and a general visuomotor skill learning measure (GSL). Gray matter volume (GMV) increased, and white matter volume (WMV) decreased from day 1 to day 2 in cerebellar areas regardless of group. A moderating effect of group was found on the correlation between WMV underlying the left precentral gyrus at day 2 and the change in ISL performance, suggesting the use of different underlying learning mechanisms in DD and TD children during the ISL task. Moreover, DD had larger WMV in the posterior thalamic radiation compared with TD, supporting previous reports of atypical development of this structure in DD. Further studies with larger sample sizes are warranted to validate these results.

## Introduction

Developmental dyslexia (DD) is defined as experiencing difficulties in decoding written words, even in the absence of intellectual disability and with adequate educational guidance. DD has an estimated prevalence of 5–17%, with high variability stemming from different assessment methods, as well as linguistic and socio-cultural factors^1^. DD is neurobiological in origin, yet still little is known regarding the brain structural correlates of DD. Largely conflicting reports exist concerning neuroimaging findings of structural brain differences in dyslexic individuals compared with typically developing (TD) controls^2^, with the exception of consistently smaller total intracranial volumes in dyslexic compared with controls^2^. A recent meta-analysis suggested that the focus on different writing systems across studies may have partly played a role in these inconsistencies^3^. For example, DD showed smaller gray matter volume (GMV) compared with TD controls in temporoparietal, occipitotemporal, and cerebellar cortices in alphabetic languages^3,4^, while the GMV of the left inferior frontal gyrus was more affected in morpho-syllabic languages^3^. Alterations in the temporo-parietal junction, in particular, may be a potential marker of the risk for developing dyslexia in pre-readers^5^.

Alterations of the cerebellum and temporo-parietal structures can also be observed in the underlying white matter^6–8^. White matter abnormalities in DD, however, extend well beyond thalamocortical projections and the reading network^9,10^, encompassing also the limbic system and the motor system, especially the cerebellar fibers and corona radiata^9^. The arcuate fasciculus seems to be particularly relevant to the development of dyslexia^8,11,12^. This tract connects the temporal cortex and inferior parietal cortex to the frontal lobe, and is responsible for connecting Broca’s and Wernicke’s areas, as well as connecting the visual word form area to language related areas, such as the planum temporale^11,12^.

While most previous studies have focused on the processes subserving reading and writing abilities, other deficits have been observed in individuals with DD. For example, studies using the Serial Reaction Time (SRT) task^13^ have reported implicit sequence learning deficits in children^14–17^ and young adults with DD^18–20^, in contrast with well-preserved explicit learning abilities^15,21,22^.

The SRT task is a complex task encompassing several different cognitive functions, involving procedural learning and the acquisition and use of complex, sequence-based motor, perceptual and cognitive skills^23^. The task resembles a four-choice reaction time task. A visual cue appears at any of four positions on a computer screen. When a cue appears, the participant has to select the corresponding response button. Sequential trials, in which the cues follow repeating sequence of positions, are alternated with random trials, in which the cues don’t follow any repeating patterns of positions^24^. In addition to the implicit recognition and prediction of sequential associations (i.e., sequence-specific learning, henceforth ISL), the task also requires the integration of visual and motor responses, and fine movement control, that is, a more general visuomotor skill learning (henceforth GSL)^25^. ISL and GSL are subserved by partly different brain structures. Basal ganglia seem to sustain ISL, while additional structures are recruited for GSL, including the premotor cortex and cerebellum^25^.

The impairment in ISL observed in DD has been suggested to be task-dependent, and more evident when performing higher-order sequence learning tasks^14,18,19,21,26^. Moreover, it seems to be more evident after extended practice over repeated training sessions, during the consolidation phase and subsequent stages of learning^15,27^. Whether this may reflect potential hindrances in short-term plasticity of the involved brain structures is, however, yet to be explored, as no studies have been conducted so far to explore the neurobiological underpinnings and brain plasticity effects of repeated ISL practice in DD.

Recent studies employing repeated structural imaging of the adult human brain, in fact, have demonstrated that structural brain changes, measured as changes in estimated local GMV and WMV, can be induced by motor skill learning^28,29^. Such morphological brain changes can be detected already after a few days^30,31^ or even minutes of practice^32,33^, and seem to be particularly prominent in the motor cortex^33–35^ and cerebellar structures^33,36–38^.

The aim of the present study was therefore to examine potential atypicalities in short-term learning-related brain plasticity in DD by examining GMV and WMV changes related to ISL and GSL on the SRT task in a group of children with DD and TD control children. Recently, we examined the neural correlates of sequence-specific learning on the SRT task in children with DD compared to TD controls^39^. The study was performed over two days with a 24-h inter-session interval. We expected that SRT training would produce less brain plasticity changes in children with DD compared with TD children, mainly reflected by less pronounced post-training increases in GMV in relevant brain areas (suggestive of neurogenesis, synaptogenesis and production of non-neuronal supportive cells). We also expected the extent of WMV modifications, reflective of myelin changes, to be smaller in DD children compared with TD.

## Results

### Behavioral measures

**Fig. 1:**
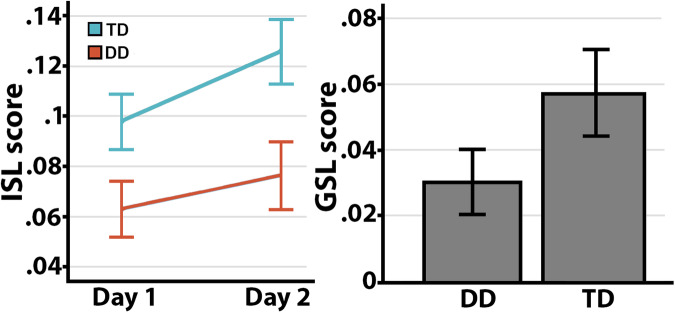
ISL and GSL scores. The figure represents the mean implicit sequence learning (ISL; left panel) and general skill learning (GSL; right panel) scores at day 1 and day 2 in children with developmental dyslexia (DD) and typically developing children (TD). Overall, both groups improved on their ISL score with the same pattern; however, TD children consistently performed better than DD at both time-points. No statistically significant group differences were observed on the GSL score between DD and TD at either time-points. Error bars represents standard error of the mean (SEM).

Behavioral results have been reported and described in detail previously^39^. Briefly, there was a trend for the DD group to have longer average response time on the task across sessions (*p* = 0.092). GSL was observed across both groups in the form of a significant reduction in reaction times from day 1 to day 2 (*p* < 0.001), but there were no group differences in GSL (*p* = 0.12). The DD group showed significantly less ISL at both days; however, the groups did not differ in terms of ISL change from day 1 to day 2 (*p* = 0.390; Table 1, Fig. 1). In summary, performance differences between groups were specific to ISL, while no differences in GSL were observed.

**Table 1:** Performance on the ISL task

	*DD* *Mean (SD)*	*TD* *Mean (SD)*	*B*	*P*	*95% C.I*.
*General RT performance*					
RT day 1	390.71 (47.40)	360.89 (69.83)	29.817	0.151	−11.475; 71.109
RT day 2	378.71 (44.03)	340.62 (67.20)	38.095	0.057	−1.237; 77.426
RT change (GSL)	0.0305 (0.04)	0.0574 (0.06)	−0.271	0.116	−0.007; 0.061
*Implicit sequence learning*					
ISL day 1	0.06 (0.05)	0.10 (0.05)	−0.035	0.031	−0.067; −0.003
ISL day 2	0.08 (0.06)	0.13 (0.05)	−0.049	0.012	−0.087; −0.012
ISL change	0.01 (0.05)	0.03 (0.05)	−0.014	0.390	−0.048; 0.019

*C.I.* confidence intervals, *SD* standard deviation.

### Time and group difference on GMV and WMV

**Fig. 2:**
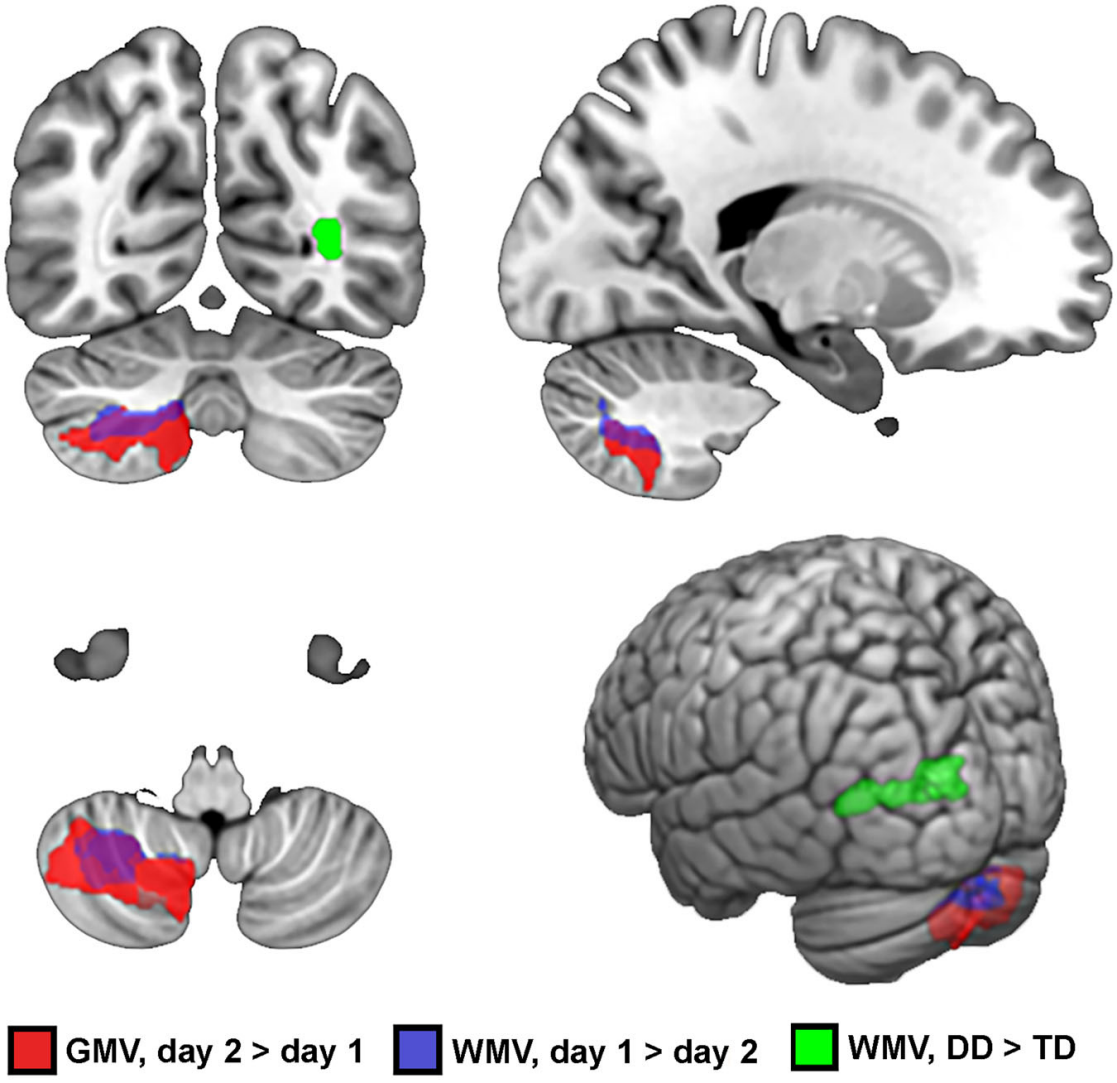
Group and time effects on GMV and WMV. The figure shows the clusters where statistically significant time and group effects were found on GMV and WMV. GMV (red cluster) was increased at day 2 compared with day 1 in the right cerebellar lobule VIII, while WMV was decreased at day 2 in the same region (purple cluster). Moreover, an effect of group was found on WMV underlying the left posterior thalamic radiation, with larger WMV in DD compared with TD (green cluster). DD developmental dyslexia, GMV gray matter volume, TD typically developing, WMV white matter volume.

A statistically significant effect of time on GMV was found in the right cerebellar lobule 8, extending to the lobule 7b and adjacent crus, exhibiting increased GMV on day 2 compared with day 1 (p FWE-corr < 0.001; t = 4.68; Cohen’s d = 0.605; Table 2, Fig. 2). No statistically significant effects of group or group × day interaction were detected, indicating that GMV changes over time occurred in both groups with a similar pattern.

**Table 2:** Effects of time and group on gray and white matter volume

p FWE-corr	C_e_ (vox)	F	MNI coordinates	Structure
*GMV* *Effect of time (Day 2* *>* *Day 1)*				
<0.001	2 612	21.92	30, −63, −48	R cerebellar lobule 8
*WMV* *Effect of time (Day 1 > Day 2)*				
<0.001	1 165	24.83	33, −57, −51	R cerebellar lobule 8
*Effect of group (DD* > *TD)*				
0.002	926	24.75	−34, −62, 6	L posterior thalamic radiation

*C*_*e*_ cluster extent (number of voxels), *DD* developmental dyslexia, *FWE* family-wise error, *L* left, *R* right, *SMA* supplementary motor area, *TD* typically developing children.

WMV was, on the other hand, reduced on day 2 compared with day 1 (p FWE-corr = 0.001, t = 4.98, Cohen’s d = 0.617) in the right cerebellum, particularly in the right lobule 8. A main effect of group was also detected, with DD having greater WMV in the left posterior thalamic radiation and inferior longitudinal fasciculus (ILF) compared with TD, particularly in the cuneus (p FWE-corr = 0.004; t = 4.97; Cohen’s d = 1.404). No effect of the group × day interaction on WMV was found.

### Correlations between ISL, GSL, and imaging measures

**Fig. 3:**
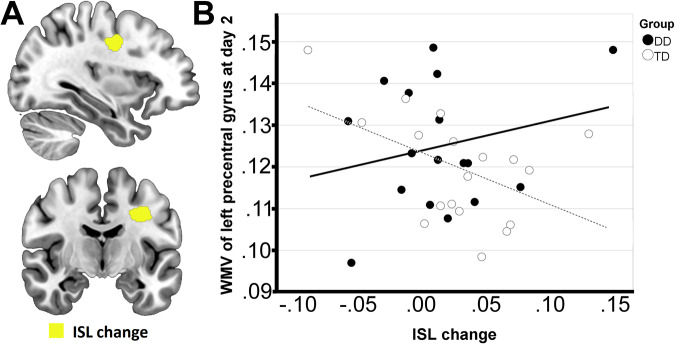
Correlations between WMV at day 2 and behavioral measures. **A** Shows the clusters where statistically significant correlations were found between WMV at day 2 and ISL change (yellow cluster). The scatterplot in **B** shows the moderating effect of group on the correlation between WMV and ISL change. While a negative correlation was present with WMV of the left precentral gyrus at day 2 in TD, no statistically significant correlation was detected in DD. DD developmental dyslexia, ISL implicit sequence(-specific) learning, TD typically developing children, WMV white matter volume.

No correlations between the change in GMV and WMV from day 1 to day 2, and ISL or GSL change, were detected. However, an effect of the group × ISL change interaction, indicating a moderating effect of group on the association between ISL change and WMV, was found on the WMV at day 2 in a cluster of 744 voxels underlying the left precentral gyrus (p FWE-corr = 0.006; F = 32.13) (Fig. 3A). In particular, while a negative correlation was present in the TD group (*p* = 0.034; r = −0.501), no correlation was observed in the DD group (*p* = 0.364, r = 0.235) (Fig. 3B). No correlations between GMV at day 2 and ISL change were detected, nor any effect of the group × ISL change interaction. No correlations between either GMV or WMV at day 2 and GSL were detected, nor any effect of the group × GSL change interaction.

## Discussion

We investigated brain plasticity in a group of children with DD, compared with TD children, after two sessions of training on a SRT task with a 24-h interval. GMV was increased at day 2 in the right cerebellar lobules 8 and 7b in the whole sample, indicating that GMV changes over time occurred in both groups with a similar pattern. WMV in the same areas was, on the other hand, reduced on day 2 compared with day 1. Moreover, DD had larger WMV in the left posterior thalamic radiation and ILF compared with TD, at both days. Furthermore, WMV underlying the left precentral gyrus at day 2 showed a positive correlation with ISL change in DD, but a negative correlation in TD.

The cerebellum is part of a larger fronto-striatal-cerebellar circuitry recruited during ISL tasks^18^. Within this network, the cerebellum has been suggested to subserve general visuomotor abilities^25^. The increase in GMV in the cerebellum from day 1 to day 2 observed in our sample is consistent with current models of skill learning, postulating the occurrence of plasticity phenomena involving neurogenesis and gliogenesis^40^. Learning-related GMV increases have been, in fact, ascribed to the interplay of different mechanisms, primarily changes in synapse density and dendritic spine morphology, and proliferation of neurons and non-neuronal cells^40^. The latter include proliferation of astrocytes, involved in supporting synaptic function, ion homeostasis, neuronal energy expense and regulating blood flow in response to neuronal activity; and microglia, which plays a role in supporting structural and functional plasticity of synapses and dendrites during both development and learning^40^. The reduction in WMV observed in our sample following skill learning is, on the other hand, of more difficult interpretation^40,41^. Plasticity changes in white matter may stem from variation in myelination, axon diameter, fiber density, or fiber geometry^40^. Most of the studies focusing on white matter plasticity during skill learning focused on microstructural diffusivity properties rather than volume^41^, leading to somewhat conflicting findings. In adults, several studies have reported increased fractional anisotropy or decreased mean diffusivity (suggestive of increased myelin volume or increased packing density of the myelin fibers) over time after motor skill learning; however, other equally valid studies have reported opposite results (for a review, see ref. ^41^). For example, training on a whole-body balancing task can induce decreases in fractional anisotropy^42^, and possibly a reduction in WMV^41^. Current evidence suggests that white matter plasticity processes following skill learning can result in both increases and decreases in WMV, such as the WMV decrease observed in our sample, depending on the duration of the intervention and the type of skill learning protocol employed^41^. The lack of group differences in GMV and WMV of this area is, on the other hand, consistent with the observation that GSL occurred to similar extent in both groups in our sample. It is worth noting, nonetheless, that alterations in cerebellar functions have been postulated to be driving the automaticity deficits observed in children with DD^43–45^. The cerebellum is, in fact, activated in healthy individuals both when executing a previously learned (automatic) sequence of finger presses, as well as when learning a new sequence of presses by trial and error^46^. People with DD, however, show significantly less cerebellar activation in both conditions^43^, along with impaired implicit motor learning compared with controls^47^. Moreover, developmental differences in cerebellar asymmetry and gray matter volume have been reported in children with DD^47,48^. However, whether the changes in cerebellar GMV and WMV in our sample were primarily driven by the improvement on general, sequence-independent visuomotor learning rather than by implicit skill learning, is difficult to interpret. Further studies including a control group performing a visuomotor task with no sequence learning involved are warranted to disentangle the effects of sequence-independent and sequence-dependent learning in the cerebellum.

The volume of the white matter underlying the left precentral gyrus at day 2 showed a trend for a positive correlation with ISL change in DD, but a negative correlation in TD. A pattern of correlations similar to ours has been previously reported in a study investigating cerebral blood flow during a SRT task in adults with schizophrenia^49^; patients showed a positive correlation between cerebral blood flow in the premotor areas (including the precentral gyrus) and learning, while a negative correlation was found in controls^49^. Functional dynamic connectivity between the precentral gyrus and the dorsal caudate has also been previously reported to be negatively correlated with performance improvement on an implicit probabilistic sequence learning (IPSL) task in adults^50^, such that participants with greater connectivity between these regions showed less improvement^50^. The different patterns of correlation suggest that different strategies are employed by the two groups to achieve performance improvement on the ISL task^49^. A lower baseline score on the ISL task may reflect a reduced ability, in the DD group, to detect a sequence (signal) that is embedded in a stream of random noise^49^. A reduced ability to infer abstract relationships between events, and to predict the occurrence of specific events from context may lead these children to rely more on explicit, stimulus-driven strategies to perform the task^49,51^. Interestingly, the right precentral gyrus has also been speculated to be involved in the pathogenesis of dyslexia independently of the disordered reading experience, as reduced GMV in this area has been reported in dyslexic children compared with age-matched controls, but also with younger controls matched on reading abilities^52^.

In our sample, however, an effect of group was only found on WMV in the left posterior thalamic radiation (extending to the ILF), which was larger in DD compared with TD, at both assessment days. The posterior part of the thalamic radiation connects the caudal parts of the thalamus with the parietal and occipital lobes via the posterior thalamic peduncle and posterior limb of the internal capsule (PLIC). A previous study performed in young adults has reported alterations in the posterior part of the thalamic radiation in a sample of young adults with DD, though pointing toward a reduction of the structural connectivity of this tract in young adults with DD compared with TD controls^53^, in contrast with our findings. This discrepancy may be driven by the different age range of the participants recruited in our study. In TD children, in fact, the PLIC, encompassing the posterior thalamic radiation, shows age-related increases in myelination and/or axonal density, reflected by increases in fractional anisotropy (FA) and decreased in apparent diffusion coefficient (ADC)^7,54^. This pattern is accentuated in children with DD, who show even higher FA values and lower ADC values than TD children up until 11 years of age^7^, followed by a decrease to control levels. The apparent diffusivity coefficient of the PLIC has also been reported to correlate with writing abilities in children with DD^55^. However, conflicting findings exist, as other studies have reported reduced FA in this tract in children with spelling difficulties compared with controls^56^, and higher FA in this tract has been related to better reading abilities^57,58^. Reading abilities have also been related to structural integrity of the ILF^59–61^, though opposite findings of a correlation between reduced FA in the ILF and better reading abilities in TD children who are just beginning to read have also been reported^62^. Future studies with larger sample size, allowing an age-based stratification of the children, might shed light on the involvement of the PLIC in dyslexia, and on the specific deviations from the normal white matter development occurring in this period of complex brain development.

The present study has limitations that have to be acknowledged. The sample size was relatively small, calling for further studies with larger sample sizes to validate our findings. Moreover, a total of 26/35 children (DD, *n* = 13/17; TD, *n* = 13/18) included in this study had participated in a previous sequence learning study using the alternating serial reaction time paradigm (Hedenius et al.^27^), and were therefore not naïve to the study task. This may have potentially limited the power to detect differences in structural brain plasticity patterns between DD and TD. It must also be mentioned that the test-retest reliability of the SRT task has been argued to be sub-optimal^23^; however, this issue impacts primarily longitudinal measures of individual differences across sessions, potentially leading to the detection of false positive improvements on the task^23^. This may have limited the power for finding correlations between changes in imaging measures and change on the task performance. On the other hand, the reliability seems to be appropriate from within-session group comparison^23^. Therefore, our finding of consistently lower within-session ISL in the DD group, compared to TD, at both days, is unlikely to be driven by the low test-retest reliability of the task. In our study, the test-retest reliability of the SRT measures was 0.6 (moderate reliability). Nonetheless, the observed improvement in performance observed from day 1 to day 2 across groups, needs to be interpreted with caution. Similar considerations must also be made concerning the lack of a non-experimental control group of children (i.e., children scanned 24 h apart without SRT training), which may raise concerns relative to the impact of measurement error on MRI findings. While intra-scanner test-retest reliability of brain volumetric measurements has been reported to be excellent in clinical and non-clinical populations^63–65^, future studies including a non-experimental group should be performed to account for repeated MRI measurement error.

In sum, we investigated brain plasticity in a group of children with DD, compared with TD children, after two sessions of training on a SRT task with a 24-h interval. GMV was increased, while WMV was reduced in the right cerebellar lobules 8 and 7b at day 2 compared with day 1 in the whole sample, suggesting the occurrence of plasticity phenomena induced by ISL practice. However, longitudinal studies are needed to evaluate the time-course and maintenance of these plasticity changes. WMV underlying the left precentral gyrus at day 2 showed a trend for a positive correlation with ISL change in DD, but a negative correlation in TD, pointing toward the use of different underlying learning mechanisms in DD and TD children for solving the ISL task, with the DD group potentially relying more on explicit, stimulus-driven strategies to compensate for a reduced ability for implicit learning. Moreover, DD had larger WMV in the left posterior thalamic radiation and ILF compared with TD, supporting previous reports of atypical development of this structure in children with DD. However, further studies with larger sample sizes, and with subjects naïve to the task to be performed, are warranted to validate and generalize these results.

## Methods

### Participants

The participating children (DD *n* = 17; TD *n* = 18) were all part of a larger behavioral study focusing on learning and memory in children with reading difficulties (The REMEMBR project), and the sample and behavioral paradigm have been described in detail in previous reports from this project^27,39^. The study was conducted in accordance with the Declaration of Helsinki, and was approved by the ethical review board of Uppsala, Sweden. All parents or legal guardians gave written informed consent, and all children provided written assent to participate in the study.

The groups did not differ with respect to age (9–13 years), sex, performance IQ (PIQ), or a language composite score based on vocabulary and syntactic comprehension (Table 3). Significant group differences were observed in word reading, reading fluency, spelling, and phoneme awareness (see ref. ^39^). All children were mono-lingual Swedish speaking, with equivalent exposure to English as a second language in school.

**Table 3:** Participant characteristics

Variable	DD (*n* = 17)		TD (*n* = 18)		Comparison	
	*M*	*SD*	*M*	*SD*	*t*	*p*
Age	12.3	0.78	12.1	1.5	0.59	0.557
Sex (F/M)	7/10		10/8		*χ*^*2*^ = 0.72	0.395
PIQ	101.5	11.7	108.3	14.6	1.51	0.141
Nonword read	7.2	5.2	63.6	23.3	9.45	<0.001
Word read	6.3	2.9	64.2	22.1	9.56	<0.001
Read fluency	1.9	0.8	4.9	1.5	7.11	<0.001
Spelling	10.9	8.6	74.5	20.8	11.23	<0.001
PA	17.5	7.3	35.7	9.3	6.26	<0.001
LangComp	6.1	1.2	5.8	1.2	0.88	0.385

*DD* Children with Developmental Dyslexia, *TD* Typically developing children, *PIQ* scores from WISC-IV performance IQ subtests (Wechsler^67^), *Nonword read* percentile scores from the nonword reading subtest from LäST (Elwér et al.^66^), *Word read* percentile scores from the word reading subtest from LäST, *Spelling* percentile scores from the spelling subtest from LäST, *Read fluency* stanine scores from the reading fluency subtest from DLS ([Diagnostic Reading and Spelling test], Järpsten & Taube^68^), *PA* raw scores from the Paulin phoneme awareness test (Joel and Berggren^78^), *LangComp* a composite score based on stanine scores from DLS vocabulary subtest (Järpsten & Taube^68^) and the Test for Reception of Grammar – 2 (TROG – 2, Bishop^69^).

Children with DD were recruited from speech and language therapy clinics in the Stockholm-Uppsala area in Sweden. Inclusion criteria were: a clinical diagnosis of DD from a certified speech and language therapist, and a word reading score <15th percentile on a standardized Swedish word reading test^66^. Exclusion criteria for the DD group were PIQ scores <80^67^, any other known comorbid neuropsychiatric condition (as reported by parents) and a language composite stanine score <3. As previously described in Hedenius and Persson^39^, the language composite score was derived from the vocabulary subtest from the DLS^68^, and the Swedish version of the Test for Reception of Grammar – 2 (TROG – 2)^69^. These DD inclusion/exclusion criteria are consistent with the Diagnostic and Statistical Manual of Mental Disorders (5th ed.; DSM-5)^70^ as well as with previously published studies on DD (e.g., ref. ^71^).

TD children were recruited from schools in the same area. Inclusion criteria for the TD group were normal language, reading and writing development as reported by parents. Exclusion criteria were any known neurodevelopmental condition (as reported by parents), PIQ scores <80^67^, word reading, non-word reading, or spelling scores^66^ <the 20th percentile, or a language composite stanine score <3.

Possible unrecognized ADHD was ruled out using the executive functions subdomain in the Five-to-Fifteen (FTF) parent questionnaire^72^. The FTF targets ADHD symptoms, and its common co-morbidities, in children and adolescents between 5 and 15 years of age. It has been shown to be a reliable and valid screening instrument that correlates significantly with other ADHD questionnaires, as well as performance-based measures^73,74^. No child in the sample had significant ADHD symptoms.

### Implicit sequence learning

**Fig. 4:**
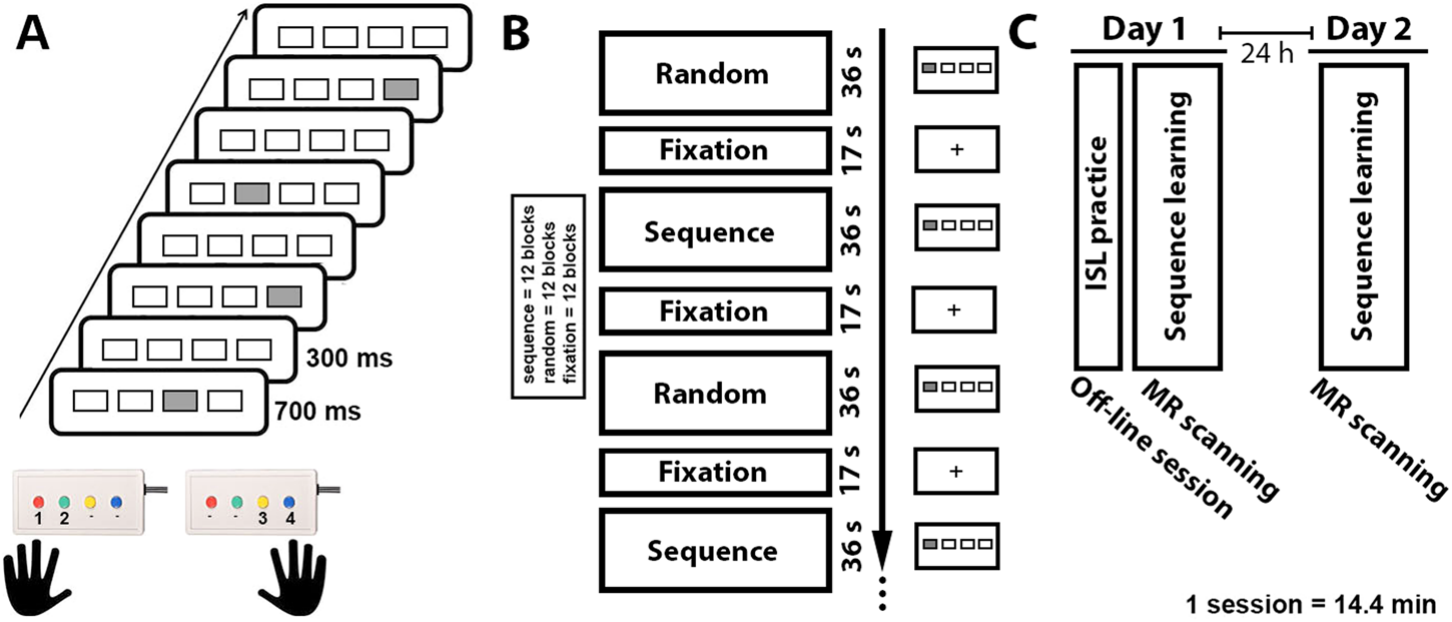
Study protocol. The figure provides a schematic overview of (**A**) the serial reaction time task used in the study; **B** the study design; **C** the collection timeline across the two days. The practice session consisted of 10 min of performance on the ISL. Behavioral data were included from the two in-scanner sessions at day 1 and 2.

Four squares were presented horizontally in the center of a computer screen. Each square position corresponded to one of four buttons, in order from left to right. Participants were instructed to press the corresponding button using the index and middle finger of each hand as quickly and accurately as possible when a white square turned gray (Fig. 4A). Behavioral data on the ISL were collected during fMRI scanning. Response accuracy and reaction times (RT) were recorded with two MRI-compatible response boxes, one for each hand. Button presses were recorded using E-prime 2.0^75^. The task was administered in two sessions on two separate days (day 1 and day 2), with a 24-h inter-session-interval (Fig. 1C). Each session included 24 blocks. Each block consisted of 36 trials, and each trial lasted 700 milliseconds (ms) with a 300 ms inter-stimulus interval. In half of the blocks, and unknown to the participants, the trials followed a fixed second-order 12-item sequence with positions from left (1) to right (4) of 1–2–1–4–2–3–4–1–3–2-4–3^76^. In the remaining blocks, trials were presented in a pseudo-random order with the constraint that two consecutive trials were not the same. Sequence and random blocks were alternated, and each block was separated by a 17-second fixation period (Fig. 1B). Error trials or omissions were excluded from analysis and median response times were used to minimize the influence of outlier responses.

### MRI acquisition

Structural images were acquired 24 h apart on Discovery^TM^ MR750 3.0 Tesla scanner, with a 32-channel phased array receiving head coil (General Electric). T1-weighted 3D spoiled gradient recalled (SPGR) images were acquired, with 0.94 × 0.94 × 1 mm^3^ voxel size (TR: 7.908 ms, TE: 3.06 ms, field of view: 24 cm, 176 axial slices, flip angle of 12).

### Preprocessing of MR images

Preprocessing of structural images was performed with CAT12 (https://neuro-jena.github.io/cat/), with the longitudinal processing pipeline optimized for the detection of subtler changes in response to short-term plasticity effects (https://neuro-jena.github.io/cat12-help/). Prior to applying the CAT12 longitudinal pipeline, customized tissue probability maps were created for our pediatric sample. To this purpose, an initial segmentation of the images was performed using Statistical Parametric Mapping 12 (SPM12) (https://www.fil.ion.ucl.ac.uk/spm/software/download/), running on Matlab 2022b. The segmentation maps were then fed to the Template-O-Matic (TOM8) toolbox (https://neuro-jena.github.io/software.html#tom), for the creation of the customized template. An average template was fitted from the segmented tissue maps, including age (modeled with a third order, cubic regression) and gender as regressors. The customized tissue probability maps generated with TOM8 were used for the longitudinal segmentation pipeline implemented in CAT 12.

In brief, the CAT 12 longitudinal pipeline consists of a preliminary inverse-consistent rigid-body registrations to realign all images for each participant, followed by the application of intra-subject bias-field corrections. The images are then segmented individually into gray matter (GM), white matter and cerebrospinal fluid. The customized tissue probability maps obtained with TOM 8 were used for segmentation. For each participant, a mean spatial transformation for all time-points is then calculated for spatial registration to the standard Montreal Neurological Institute (MNI) brain template. These mean deformations are then applied to individual images, to obtain normalization to the MNI space. Finally, smoothing with a 6 mm FWHM Gaussian kernel was applied.

Data quality was checked by estimating sample homogeneity measures. Data that deviate from the sample increase variance and can negatively affect statistical power. The mean Z-score measures the homogeneity of the final data, reflecting the quality of the images after preprocessing; a low Z-score reflects poor data quality. The image quality rating (IQR), on the other hand, combines measurements of noise and spatial resolution of the images before pre-processing. The product between IQR * mean Z-scores was used to evaluate data quality, as recommended by CAT 12 manual. Three participants were flagged based on the IQR*mean Z-score product; two participants had low Z-score (deviation from the sample after pre-processing), while one participant had high IQR (deviation from the sample before pre-processing). Visually inspecting the data for the presence of artifacts or low image quality is recommended in such cases, to confirm whether the subject is an outlier. If no artifacts are detected and the image quality is appropriate, the data can be retained (https://neuro-jena.github.io/cat12-help/#module4). Visual inspection of the un-preprocessed and pre-processed data did not show any artifacts or issues with data quality; therefore, the data were retained in the analysis. Nonetheless, all analyses were also performed without these potential outliers, leading to the same results.

### Statistical analysis

Statistical analysis of behavioral data was performed using Statistical Package for Social Sciences (SPSS). For each participant, we calculated the median RT for the random and sequence blocks of each session, separately. General skill learning (GSL) was defined as the sequence-independent RT decrease from day 1 to day 2, and was calculated from the average RT across sequence and random trials. Sequence-specific learning (ISL) was operationalized as the median RT difference, for each session, between random and sequence blocks. Because longer average response times will lead to numerically larger differences (and thus erroneously to more “learning” in the slower group) for both the GSL and ISL measures, a RT normalizing procedure was used. The GSL measure was normalized by dividing the RT difference between day 1 and day 2 with the average RT across both days. For the ISL measure, we followed the procedure outlined in Hedenius et al.^77^ to calculate a normalized sequence learning measure. This measure was obtained by dividing the difference between the median RTs for the random and sequence blocks, in each session, by the average median RT across both random and sequence blocks, for that same session (i.e. (median RT for random blocks in session X - median RT for sequence blocks in session X)/((median RT for random blocks in session X + median RT for sequence blocks in session X)/2). For both measures, larger numbers reflect more learning.

The following variables were used as behavioral outcome measures for the SRT task: (1) the difference in ISL between day 1 and day 2 (henceforth ISL), reflecting the amount of sequence-specific learning from day 1 to day 2; (2) the change in the average RT across sequence and random trials between day 1 and day 2 (henceforth GSL), reflecting the amount of general skill learning from day 1 to day 2. Behavioral measures were tested for normality of distribution with the Shapiro Wilk’s test for normality. No outliers were detected in either measurement. Separate ANOVA tests were used to test for between-groups differences in baseline ISL and GSL, ISL and GSL changes from day 1 to day 2. The threshold for significance was set at *p* < 0.05.

Statistical analysis of the imaging data was performed with CAT12. A flexible factorial design for longitudinal data was used. The flexible factorial model allows for mixed-model specification, with group set as between-subject factor (two levels: DD, TD), and time set as within-subject factor (two levels: day 1, day 2). Main effects of time and group, and the effect of the group × day interaction, were tested. A preliminary uncorrected threshold of *p* < 0.001 was applied. Voxels surviving such threshold were further corrected for family-wise error (FWE) rate at cluster level with a threshold of *p* < 0.05. The same analyses were performed on gray matter volume (GMV) and white matter volume (WMV).

GMV and WMV change from day 1 to day 2, and GMV and WMV at day 2 were tested for correlations with the ISL change from day 1 to day 2 (reflective of sequence-specific learning), and with GSL (reflective of visuomotor skill learning over training sessions). Voxel-wise, whole-brain correlation analyses were carried out in CAT12. Group was included as a potential moderator for the correlation between imaging and behavioral measures in all analyses, by testing for an interaction effect of group × ISL change, and group × GSL. A preliminary uncorrected threshold of *p* < 0.001 was applied. Voxels surviving such threshold were further corrected for family-wise error (FWE) rate at cluster level with a threshold of *p* < 0.05.

### Reporting summary

Further information on research design is available in the Nature Research Reporting Summary linked to this article.

### Supplementary information

Reporting summary

## Data Availability

The data that support the findings of this study are available on request. The data are not publicly available due to GDPR restrictions. Data can be requested to J.P. A Data Use Agreement will be requested.
