# Supplementary material for: Exploring brain plasticity in developmental dyslexia through implicit sequence learning
Source: NPJ Sci Learn. 2024 May 27;9:37. doi: 10.1038/s41539-024-00250-w (PMC11130236; doi:10.1038/s41539-024-00250-w)
Supplement: Supplementary file 1 — Reporting summary [file 41539_2024_250_MOESM1_ESM.pdf]

nature portfolio

Corresponding author(s): Gaia Olivo, gaia.olivo@psy.gu,se

Last updated by author(s): 2024-04.24

Reporting Summary

Nature Portfolio wishes to improve the reproducibility of the work that we publish. This form provides structure for consistency and transparency in reporting. For further information on Nature Portfolio policies, see our Editorial Policies and the Editorial Policy Checklist.

Statistics

For all statistical analyses, confirm that the following items are present in the figure legend, table legend, main text, or Methods section.

n/a	Confirmed	
☐	☒	The exact sample size (*n*) for each experimental group/condition, given as a discrete number and unit of measurement
☐	☒	A statement on whether measurements were taken from distinct samples or whether the same sample was measured repeatedly
☐	☒	The statistical test(s) used AND whether they are one- or two-sided<br>*Only common tests should be described solely by name; describe more complex techniques in the Methods section.*
☐	☒	A description of all covariates tested
☐	☒	A description of any assumptions or corrections, such as tests of normality and adjustment for multiple comparisons
☐	☒	A full description of the statistical parameters including central tendency (e.g. means) or other basic estimates (e.g. regression coefficient) AND variation (e.g. standard deviation) or associated estimates of uncertainty (e.g. confidence intervals)
☐	☒	For null hypothesis testing, the test statistic (e.g. F, t, r) with confidence intervals, effect sizes, degrees of freedom and P value noted<br>*Give P values as exact values whenever suitable.*
☒	☐	For Bayesian analysis, information on the choice of priors and Markov chain Monte Carlo settings
☒	☐	For hierarchical and complex designs, identification of the appropriate level for tests and full reporting of outcomes
☐	☒	Estimates of effect sizes (e.g. Cohen's d, Pearson's r), indicating how they were calculated

Our web collection on statistics for biologists contains articles on many of the points above.

Software and code

Policy information about availability of computer code

Data collection	No customised code was used in this manuscript.
Data analysis	No code was used in this manuscript.

For manuscripts utilizing custom algorithms or software that are central to the research but not yet described in published literature, software must be made available to editors and reviewers. We strongly encourage code deposition in a community repository (e.g. GitHub). See the Nature Portfolio guidelines for submitting code & software for further information.

Data

Policy information about availability of data

All manuscripts must include a data availability statement. This statement should provide the following information, where applicable:

- Accession codes, unique identifiers, or web links for publicly available datasets
- A description of any restrictions on data availability
- For clinical datasets or third party data, please ensure that the statement adheres to our policy

The data that support the findings of this study are available on request. The data are not publicly available due to GDPR restrictions.

Research involving human participants, their data, or biological material

Policy information about studies with human participants or human data. See also policy information about sex, gender (identity/presentation), and sexual orientation and race, ethnicity and racism.

Reporting on sex and gender	The sample included in the study consists of 17 females and 18 males. Information concerning sex was self-reported. The patients group includes 7 females and 10 males. The control groups includes 10 females and 8 males. Given the small sample size, the analysis was not stratified based on sex. Sex of the participants is reported in the methods. No individual data are reported. See methods.
Reporting on race, ethnicity, or other socially relevant groupings	Race and/or ethnicity information were not collected. However, they were all Swedish mono-lingual speaking.
Population characteristics	The participating children (DD n = 17; TD n = 18) were all part of a larger behavioral study focusing on learning and memory in children with reading difficulties (The REMEMBR project), and the sample and behavioral paradigm have been described in detail in previous reports from this project (Hedenius et al., 2021; Hedenius & Persson, 2022). The study was conducted in accordance with the Declaration of Helsinki, and was approved by the ethical review board of Uppsala, Sweden. All parents or legal guardians gave written informed consent, and all children provided written assent to participate in the study.<br>The groups did not differ with respect to age (9 to 13 years), sex, performance IQ (PIQ), or a language composite score based on vocabulary and syntactic comprehension (Table 1). Significant group differences were observed in word reading, reading fluency, spelling, and phoneme awareness (see (Hedenius and Persson 2022)). All children were mono-lingual Swedish speaking, with equivalent exposure to English as a second language in school. See methods.
Recruitment	The participating children (DD n = 17; TD n = 18) were all part of a larger behavioral study focusing on learning and memory in children with reading difficulties (The REMEMBR project), and the sample and behavioral paradigm have been described in detail in previous reports from this project (Hedenius et al., 2021; Hedenius & Persson, 2022). See methods.
Ethics oversight	The study was conducted in accordance with the Declaration of Helsinki, and was approved by the ethical review board of Uppsala, Sweden. See methods.

Note that full information on the approval of the study protocol must also be provided in the manuscript.

Field-specific reporting

Please select the one below that is the best fit for your research. If you are not sure, read the appropriate sections before making your selection.

☐ Life sciences ☒ Behavioural & social sciences ☐ Ecological, evolutionary & environmental sciences

For a reference copy of the document with all sections, see nature.com/documents/nr-reporting-summary-flat.pdf

Behavioural & social sciences study design

All studies must disclose on these points even when the disclosure is negative.

Study description	Deficits in implicit sequence learning (ISL) abilities have been reported in children with DD. We investigated brain plasticity in a group of 17 children with DD, compared with 18 typically developing (TD) children, after two sessions of training on a serial reaction time (SRT) task with a 24-hours interval. Our outcome measures for the task were: a sequence-specific implicit learning measure (ISL), entailing implicit recognition and learning of sequential associations; and a general visuomotor skill learning measure (GSL). Gray matter volume (GMV) increased, and white matter volume (WMV) decreased from day 1 to day 2 in cerebellar areas regardless of group. A moderating effect of group was found on the correlation between WMV underlying the left precentral gyrus at day 2 and the change in ISL performance, suggesting the use of different underlying learning mechanisms in DD and TD children during the ISL task. Moreover, DD had larger WMV in the posterior thalamic radiation compared with TD, supporting previous reports of atypical development of this structure in DD.<br>The methods used were quantitative.
Research sample	Children with DD were recruited from speech and language therapy clinics in the Stockholm-Uppsala area in Sweden. Inclusion criteria were i) a clinical diagnosis of DD from a certified speech and language therapist, and ii) a word reading score < 15th percentile on a standardized Swedish word reading test (Elwér, Fridolfsson et al. 2011). Exclusion criteria for the DD group were PIQ scores < 80 (Wechsler 2003), any other known comorbid neuropsychiatric condition (as reported by parents) and a language composite stanine score < 3. As previously described in Hedenius & Persson (2022), the language composite score was derived from the vocabulary subtest from the DLS (Järpsten & Taube, 2010), and the Swedish version of the Test for Reception of Grammar – 2 (TROG – 2, Bishop, 2009). These DD inclusion/exclusion criteria are consistent with the Diagnostic and Statistical Manual of Mental Disorders (5th ed.; DSM-5; American Psychiatric Association, 2013) as well as with previously published studies on DD (e.g., (Knoop-van Campen, Segers et al. 2018).<br>TD children were recruited from schools in the same area. Inclusion criteria for the TD group were normal language, reading and writing development as reported by parents. Exclusion criteria were any known neurodevelopmental condition (as reported by parents), PIQ scores < 80 (Wechsler 2003), word reading, non-word reading, or spelling scores (Elwér, Fridolfsson et al. 2011) < the 20th percentile, or a language composite stanine score < 3.<br>See methods.

Sampling strategy	No power analysis was performed.
Data collection	One guardian was present during data collection. The cognitive task was computerized. Four squares were presented horizontally in the center of a computer screen. Each square position corresponded to one of four buttons, in order from left to right. Participants were instructed to press the corresponding button using the index and middle finger of each hand as quickly and accurately as possible when a white square turned gray (figure 1a). Behavioral data on the ISL were collected during fMRI scanning. Response accuracy and reaction times (RT) were recorded with two MRI-compatible response boxes, one for each hand. Button presses were recorded using E-prime 2.0 (Psychology Software Tools, Inc., 2002). The task was administered in two sessions on two separate days (day 1 and day 2), with a 24-hour inter-session-interval (figure 1c). Each session included 24 blocks. Each block consisted of 36 trials, and each trial lasted 700 milliseconds (ms) with a 300 ms inter-stimulus interval. In half of the blocks, and unknown to the participants, the trials followed a fixed second-order 12-item sequence with positions from left (1) to right (4) of 1–2–1–4–2–3–4–1–3–2-4–3 (Schendan, Searl et al. 2003). In the remaining blocks, trials were presented in a pseudo-random order with the constraint that two consecutive trials were not the same. Sequence and random blocks were alternated, and each block was separated by a 17-second fixation period (figure 1b). Error trials or omissions were excluded from analysis and median response times were used to minimize the influence of outlier responses.<br>See methods.
Timing	Data collection was performed between 2016 and 2019
Data exclusions	No data were excluded.
Non-participation	No drop-outs occurred.
Randomization	No randomization (patients vs controls).

Reporting for specific materials, systems and methods

We require information from authors about some types of materials, experimental systems and methods used in many studies. Here, indicate whether each material, system or method listed is relevant to your study. If you are not sure if a list item applies to your research, read the appropriate section before selecting a response.

Materials & experimental systems

n/a	Involved in the study
☒	☐ Antibodies
☒	☐ Eukaryotic cell lines
☒	☐ Palaeontology and archaeology
☒	☐ Animals and other organisms
☒	☐ Clinical data
☒	☐ Dual use research of concern
☒	☐ Plants

Methods

n/a	Involved in the study
☒	☐ ChIP-seq
☒	☐ Flow cytometry
☐	☒ MRI-based neuroimaging

Plants

Seed stocks	n/a
Novel plant genotypes	n/a
Authentication	n/a

Magnetic resonance imaging

Experimental design

Design type	Block design
Design specifications	Four squares were presented horizontally in the center of a computer screen. Each square position corresponded to one of four buttons, in order from left to right. Participants were instructed to press the corresponding button using the index and middle finger of each hand as quickly and accurately as possible when a white square turned gray (figure 1a). Behavioral data on the ISL were collected during fMRI scanning. Response accuracy and reaction times (RT) were

recorded with two MRI-compatible response boxes, one for each hand. Button presses were recorded using E-prime 2.0 (Psychology Software Tools, Inc., 2002). The task was administered in two sessions on two separate days (day 1 and day 2), with a 24-hour inter-session-interval (figure 1c). Each session included 24 blocks. Each block consisted of 36 trials, and each trial lasted 700 milliseconds (ms) with a 300 ms inter-stimulus interval. In half of the blocks, and unknown to the participants, the trials followed a fixed second-order 12-item sequence with positions from left (1) to right (4) See methods.of 1–2–1–4–2–3–4–1–3–2-4–3 (Schendan, Searl et al. 2003). In the remaining blocks, trials were presented in a pseudo-random order with the constraint that two consecutive trials were not the same. Sequence and random blocks were alternated, and each block was separated by a 17-second fixation period (figure 1b). Error trials or omissions were excluded from analysis and median response times were used to minimize the influence of outlier responses.

Behavioral performance measures

Button press and reaction times were recorded. Error trials or omissions were excluded from analysis and median response times were used to minimize the influence of outlier responses.

Acquisition

Imaging type(s)

Structural acquisition.

Field strength

3

Sequence & imaging parameters

Structural images were acquired 24 hours apart on DiscoveryTM MR750 3.0 Tesla scanner, with a 32-channel phased array receiving head coil (General Electric). T1-weighted 3D spoiled gradient recalled (SPGR) images were acquired, with 0.94 x 0.94 x 1 mm3 voxel size (TR: 7.908 ms, TE: 3.06 ms, field of view: 24 cm, 176 axial slices, flip angle of 12).

Area of acquisition

Whole brain.

Diffusion MRI ☐ Used ☒ Not used

Preprocessing

Preprocessing software

CAT12

Normalization

Preprocessing of structural images was performed with CAT12 (https://neuro-jena.github.io/cat/), with the longitudinal processing pipeline optimized for the detection of subtler changes in response to short-term plasticity effects (https://neuro-jena.github.io/cat12-help/). Prior to applying the CAT12 longitudinal pipeline, customized tissue probability maps were created for our pediatric sample. To this purpose, an initial segmentation of the images was performed using Statistical Parametric Mapping 12 (SPM12) (https://www.fil.ion.ucl.ac.uk/spm/software/download/), running on Matlab 2022b. The segmentation maps were then fed to the Template-O-Matic (TOM8) toolbox (https://neuro-jena.github.io/software.html#tom), for the creation of the customized template. An average template was fitted from the segmented tissue maps, including age (modelled with a third order, cubic regression) and gender as regressors. The customized tissue probability maps generated with TOM8 were used for the longitudinal segmentation pipeline implemented in CAT 12.
In brief, the CAT 12 longitudinal pipeline consists of a preliminary inverse-consistent rigid-body registrations to realign all images for each participant, followed by the application of intra-subject bias-field corrections. The images are then segmented individually into gray matter (GM), white matter and cerebrospinal fluid. The customized tissue probability maps obtained with TOM 8 were used for segmentation. For each participant, a mean spatial transformation for all time-points is then calculated for spatial registration to the standard Montreal Neurological Institute (MNI) brain template. These mean deformations are then applied to individual images, to obtain normalization to the MNI space. Finally, smoothing with a 6 mm FWHM Gaussian kernel was applied.

Normalization template

TOM 8 customised template. As reported in the methods.

Noise and artifact removal

No fMRI involved.

Volume censoring

Data quality was checked by estimating sample homogeneity measures. Data that deviate from the sample increase variance and can negatively affect statistical power. The mean Z-score measures the homogeneity of the final data, reflecting the quality of the images after preprocessing; a low Z-score reflects poor data quality. The image quality rating (IQR), on the other hand, combines measurements of noise and spatial resolution of the images before pre-processing. The product between IQR * mean Z-scores was used to evaluate data quality, as recommended by CAT 12 manual. Three participants were flagged based on the IQR*mean Z-score product; two participants had low Z-score (deviation from the sample after pre-processing), while one participant had high IQR (deviation from the sample before pre-processing). Visually inspecting the data for the presence of artifacts or low image quality is recommended in such cases, to confirm whether the subject is an outlier. If no artifacts are detected and the image quality is appropriate, the data can be retained (https://neuro-jena.github.io/cat12-help/#module4). Visual inspection of the un-preprocessed and pre-processed data did not show any artifacts or issues with data quality; therefore the data were retained in the analysis. Nonetheless, all analyses were also performed without these potential outliers, leading to the same results.

Statistical modeling & inference

Model type and settings

Statistical analysis of behavioral data was performed using Statistical Package for Social Sciences (SPSS). For each participant, we calculated the median RT for the random and sequence blocks of each session, separately. General skill learning (GSL) was defined as the sequence-independent RT decrease from day 1 to day 2, and was calculated from the average RT across sequence and random trials. Sequence-specific learning (ISL) was operationalized as the median RT difference, for each session, between random and sequence blocks. Because longer average response times will lead to numerically larger

differences (and thus erroneously to more "learning" in the slower group) for both the GSL and ISL measures, a RT normalizing procedure was used. The GSL measure was normalized by dividing the RT difference between day 1 and day 2 with the average RT across both days. For the ISL measure, we followed the procedure outlined in Hedenius et al (Hedenius, Persson et al. 2011) to calculate a normalized sequence learning measure. This measure was obtained by dividing the difference between the median RTs for the random and sequence blocks, in each session, by the average median RT across both random and sequence blocks, for that same session (i.e. (median RT for random blocks in session X - median RT for sequence blocks in session X)/((median RT for random blocks in session X + median RT for sequence blocks in session X)/2). For both measures, larger numbers reflect more learning.

Effect(s) tested

The following variables were used as behavioral outcome measures for the SRT task: (1) the difference in ISL between day 1 and day 2 (henceforth ISL), reflecting the amount of sequence-specific learning from day 1 to day 2; (2) the change in the average RT across sequence and random trials between day 1 and day 2 (henceforth GSL), reflecting the amount of general skill learning from day 1 to day 2. Behavioral measures were tested for normality of distribution with the Shapiro Wilk's test for normality. No outliers were detected in either measurement. Separate ANOVA tests were used to test for between-groups differences in baseline ISL and GSL, ISL and GSL changes from day 1 to day 2. The threshold for significance was set at $p < .05$.
Statistical analysis of the imaging data was performed with CAT12. A flexible factorial design for longitudinal data was used. The flexible factorial model allows for mixed-model specification, with group set as between-subject factor (two levels: DD, TD), and time set as within-subject factor (two levels: day 1, day 2). Main effects of time and group, and the effect of the group × day interaction, were tested. The same analyses were performed on gray matter volume (GMV) and white matter volume (WMV).
GMV and WMV change from day 1 to day 2, and GMV and WMV at day 2 were tested for correlations with the ISL change from day 1 to day 2 (reflective of sequence-specific learning), and with GSL (reflective of visuomotor skill learning over training sessions). Voxel-wise, whole-brain correlation analyses were carried out in CAT12. Group was included as a potential moderator for the correlation between imaging and behavioral measures in all analyses, by testing for an interaction effect of group × ISL change, and group × GSL.

Specify type of analysis: ☒ Whole brain ☐ ROI-based ☐ Both

Statistic type for inference
(See Eklund et al. 2016)

A preliminary uncorrected threshold of $p < 0.001$ was applied. Voxels surviving such threshold were further corrected for family-wise error (FWE) rate at cluster level with a threshold of $p < 0.05$.

Correction

A preliminary uncorrected threshold of $p < 0.001$ was applied. Voxels surviving such threshold were further corrected for family-wise error (FWE) rate at cluster level with a threshold of $p < 0.05$.

Models & analysis

n/a	Involved in the study
☒	☐ Functional and/or effective connectivity
☒	☐ Graph analysis
☒	☐ Multivariate modeling or predictive analysis
